# The Impact of Water Potential and Temperature on Native Species’ Capability for Seed Germination in the Loess Plateau Region, China

**DOI:** 10.3390/plants13050693

**Published:** 2024-02-29

**Authors:** Guifang Hu, Xinyue He, Ning Wang, Jun’e Liu, Zhengchao Zhou

**Affiliations:** School of Geography and Tourism, Shaanxi Normal University, Xi’an 710119, China; hgf200110@163.com (G.H.); hxy0406@snnu.edu.cn (X.H.); liujune5@163.com (J.L.); zhouzhengchao@126.com (Z.Z.)

**Keywords:** seed germination, temperature, water potential, thermal time model, hydrotime model, Loess Plateau

## Abstract

Global warming is increasing the frequency and intensity of heat waves and droughts. One important phase in the life cycle of plants is seed germination. To date, the association of the temperature and water potential thresholds of germination with seed traits has not been explored in much detail. Therefore, we set up different temperature gradients (5–35 °C), water potential gradients (−1.2–0 MPa), and temperature × water potential combinations for nine native plants in the Loess Plateau region to clarify the temperature and water combinations suitable for their germination. Meanwhile, we elucidated the temperature and water potential thresholds of the plants and their correlations with the mean seed mass and flatness index by using the thermal time and hydrotime models. According to our findings, the germination rate was positively correlated with the germination percentage and water potential, with the former rising and the latter decreasing as the temperature increased. Using the thermal time and hydrotime models, the seed germination thresholds could be predicted accurately, and the germination thresholds of the studied species varied with an increase in germination percentage. Moreover, temperature altered the impact of water potential on the germination rate. Overall, the base water potential for germination, but not the temperature threshold, was negatively correlated with mean seed mass and was lower for rounder seeds than for longer seeds. This study contributes to improving our understanding of the seed germination characteristics of typical plants and has important implications for the management and vegetation restoration of degraded grasslands.

## 1. Introduction

Global climate change is associated with an increase in droughts and heat waves [1,2]. Global climate change may prevent, delay, or promote species regeneration from seeds, which may have an impact on the dynamics of plant populations and the composition of vegetation [3,4,5]. The impacts of climate change on plants have been extensively studied, but less attention has been paid to how these changes may affect plant regeneration [4,6]. However, based on seed germination, environment has a significant impact on plant recruitment [7,8,9]. Seed germination is an essential life history event in plants and can be affected by a variety of environmental factors and their interactions, such as temperature, water potential, salinity, pH, and depth of burial [10,11,12,13]. The two most important environmental variables for seed germination and the ensuing growth of seedlings are thought to be temperature and water potential [14,15]. Understanding how environmental influences affect the germination of seeds can help one not only to understand and predict the ecological adaptations of species, but also to develop effective restoration strategies [16,17,18].

One major environmental element that can control enzyme activity and either stimulate or prevent the generation of hormones that influence seed germination is temperature [15,19,20,21]. The germination responses of seed lots to temperature can be characterized through three cardinal temperatures [22,23]: a base temperature (*T_b_*), below which the seeds do not germinate; an optimal temperature (*T_o_*), at which the process proceeds at its fastest pace; and a ceiling temperature (*T_c_*), above which the process is stopped [24,25,26]. These cardinal temperatures, *T_b_*, *T_c_*, and *T_o_*, are associated with the range of ecological and geographic circumstances to which a particular species has evolved, and serve to couple the timing of germination with favorable conditions for subsequent growth and development [27,28]. In general, temperate-region seeds require lower temperatures than tropical-region seeds, and wild species have lower temperature requirements than domesticated plants [29,30,31]. Understanding the germination process when affected by temperature can help in assessing the germination characteristics or the establishment potential among range species, particularly in arid and semiarid regions [32], and can be used to identify the geographical areas appropriate to a species or genotype so it can germinate and establish [33].

Another crucial environmental component for seed germination is the availability of water [14,34]. In many parts of the world, increasing global climate change is predicted to lead to an increase in aridity [35,36]. In general, the seed germination percentage declines and mean germination time increases with decreasing water potential, and certain species may retain a reasonably high germination percentage even at very low water potential [37,38]. For example, large seeds can buffer seedlings from the negative effects of drought, and they have an advantage in establishing plants under low-soil-moisture conditions [39]. Furthermore, compared to species accustomed to humid settings, those acclimated to dry habitats may be less susceptible to water stress during seed germination [15,40]. Bradford (1990) [41] developed a hydrotime model to show how decreased water potential affects the progress of seed germination, which is used to assess germination rates at various water potentials in a way that is comparable to the thermal time model [14,41]. This threshold model quantifies the hydrotime constant (*θ_H_* in MPa h^−1^), the threshold water potential for germination or inherent level of osmotic tolerance (*Ψ_b_* in MPa), and the uniformity of seed germination in the population (*σ_Ψ_*), i.e., these biological parameters reflect seed vigor [42,43]. The hydrotime model hypothesizes that germination rates, or the inverse of time to germination, are positively proportional to the degree to which the water potential of the growing medium surpasses the base or threshold value necessary for the seeds to germinate [44,45].

The most vital phase of plant development and the beginning point of a new population is thought to be seed germination [14,19,46]. In addition, it is one of the more delicate phases of life and can easily result in seed inactivation or failure of establishment [47]. For example, seed germination is driven by environmental factors (especially climatic variables), such as temperature and humidity conditions in the seedbed, and it is related to seed traits, such as size and shape [48]. Seed germination responds differently to temperature and moisture availability in different species and/or populations [49], but few studies incorporate seed traits (e.g., size, shape, etc.) when assessing the response of germination to temperature and moisture effectiveness. The shape and size of seeds have a considerable impact on the germination process and also affect the seed hardiness, vigor, and quantity of stored carbohydrates, thus affecting the success or failure of the seedling [10,50,51,52]. According to theoretical research, flat, elongated seeds should germinate more frequently than round seeds [53]. Moreover, when it comes to seed germination and seedling survival, large-seeded species frequently have an advantage over small-seeded ones [54]. Seed characteristics determine the dynamics of plant communities and shed light on how different species have adapted to environmental constraints and community structure [55].

The Loess Plateau, which is located in northern China, is one of the most severely eroded areas in the world [56] and lies in a typical arid and semiarid region [57]. The ecosystem in this region is under threat of degradation due to long-term soil erosion disturbance and drought stress, and natural vegetation regeneration and restoration are effective ways to curb ecological degradation [58]. However, the hilly–gullied Loess Plateau region is characterized by an arid climate and water deficit, which are serious constraints to vegetation restoration and ecological construction [59]. Therefore, understanding the effects of environmental factors on seed germination would be useful for conservation and restoration. The most crucial elements in seed germination and seedling establishment, according to earlier research, are temperature and water potential [60]. However, not much research has been conducted to date on the region’s base water potential and temperature threshold—also referred to as the three cardinal temperatures—for seed germination [14,61]. In addition, it is unknown how base water potential, temperature threshold, and seed size and shape relate to plant germination.

Therefore, we performed a series of laboratory experiments on nine plant species from the hilly–gullied Loess Plateau region to address the following questions: (1) What impact do temperature and water potential have individually and together on native plant seed germination? (2) What is the base water potential and temperature threshold for germination in these species? (3) How do various plants respond when it comes to temperature and water stress during germination? (4) How do temperature and base water potential thresholds for germination relate to seed traits (size, shape)? (5) What are the appropriate temperature and moisture combinations for germination in these species? In general, this study contributes to a deeper understanding of the impact of changes in climatic variables (temperature, moisture) on the seed germination of plant populations in the hilly–gullied Loess Plateau region; additionally, it increases our understanding of the ecological adaptations of species and enables us to formulate effective strategies for restoration.

## 2. Results

### 2.1. Germination Responses to Temperature

**Figure 1:**
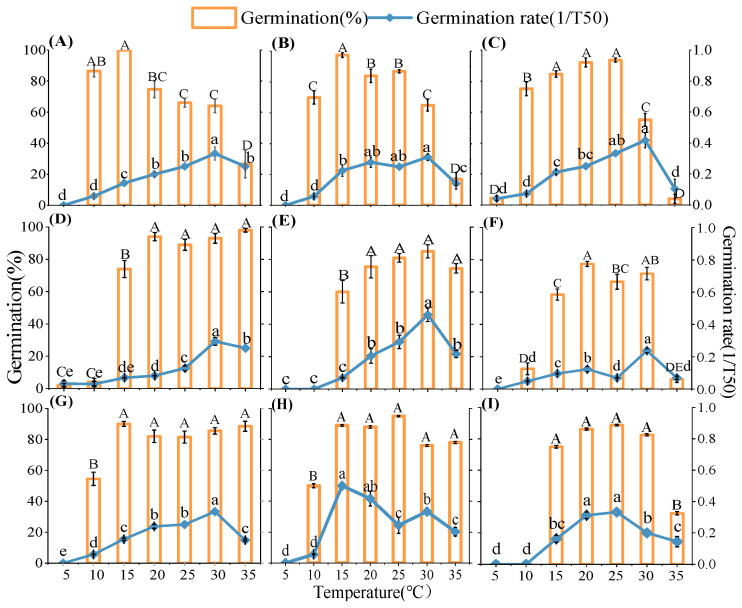
Percentage and rate (1/T_50_) of seed germination in nine species at seven different temperatures. Different uppercase letters indicate that germination percentage differed significantly (*p* < 0.05) between temperature treatments; Different lowercase letters indicate that germination rate (1/T_50_) differed significantly (*p* < 0.05) between temperature treatments; (**A**) *Artemisia scoparia*, (**B**) *Artemisia giraldii*, (**C**) *Artemisia sacrorum*, (**D**) *Periploca sepium*, (**E**) *Bothriochloa ischaemum*, (**F**) *Patrinia scabiosifolia*, (**G**) *Linum usitSatissimum*, (**H**) *Lespedeza davurica*, (**I**) *Sophora davidii*.

**Figure 2:**
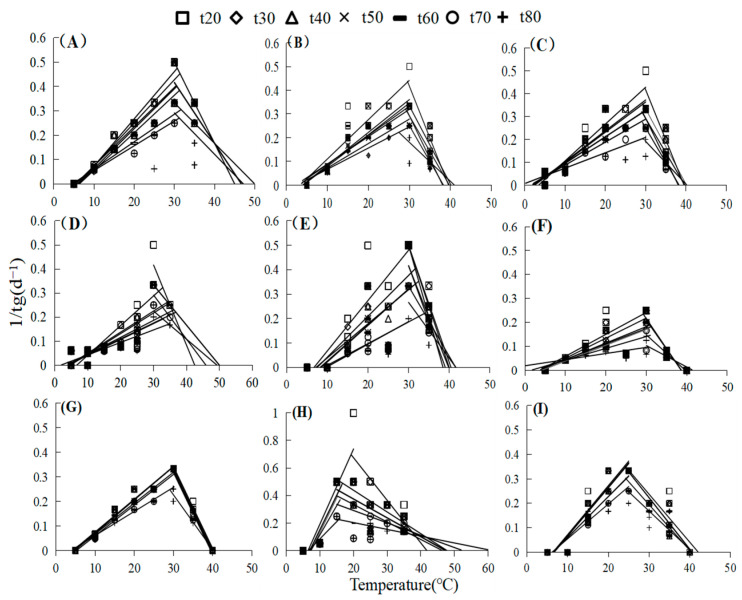
Linear regression of temperatures and percentages of germination (1/t_g_) in nine species at various percentiles where t_g_ is the time to reach a specific germination percentage (d); (**A**) *Artemisia scoparia*, (**B**) *Artemisia giraldii*, (**C**) *Artemisia sacrorum*, (**D**) *Periploca sepium*, (**E**) *Bothriochloa ischaemum*, (**F**) *Patrinia scabiosifolia*, (**G**) *Linum usitatissimum*, (**H**) *Lespedeza davurica*, (**I**) *Sophora davidii*.

**Figure 3:**
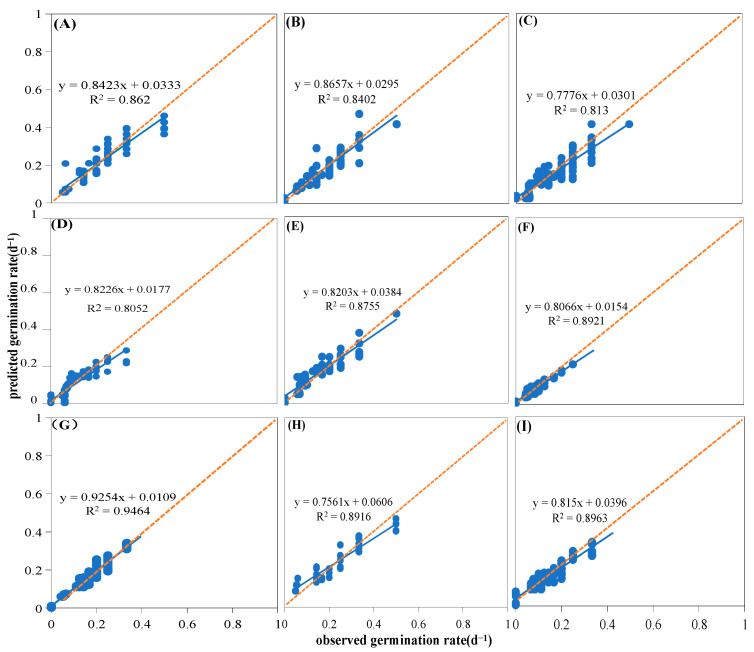
Linear fit of observed and predicted germination rates of nine species. The blue line represents the fitted trend line of the observed germination rates to the predicted germination rates; the dots represent the predicted germination rates (predicted by the thermal time model) for various observed germination rates. (**A**) *Artemisia scoparia*, (**B**) *Artemisia giraldii*, (**C**) *Artemisia sacrorum*, (**D**) *Periploca sepium*, (**E**) *Bothriochloa ischaemum*, (**F**) *Patrinia scabiosifolia* (**G**) *Linum usitatissimum*, (**H**) *Lespedeza davurica*, (**I**) *Sophora davidii*.

**Figure 4:**
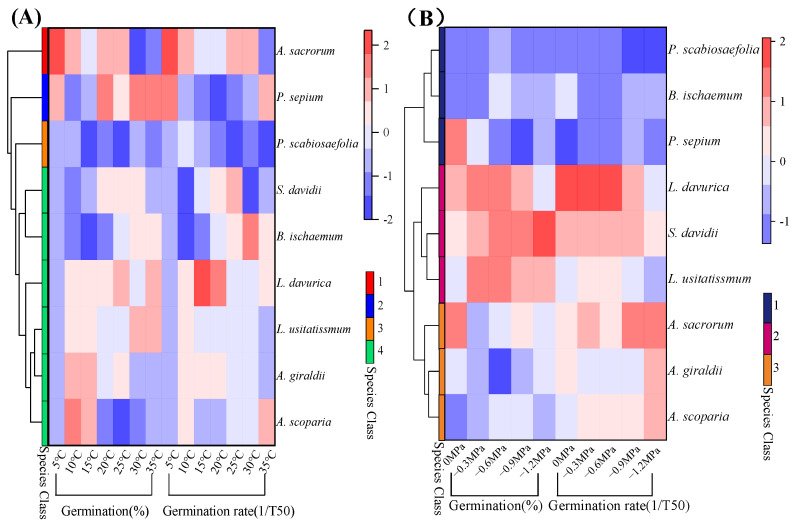
The clustering analysis of germination percentages and rates (1/T_50_) under different treatments among nine species; (**A**) germination percentages and rates (1/T_50_) for temperature treatments; (**B**) germination percentages and rates (1/T_50_) for water potential treatments.

The germination rate and percentage of nine native plant species from the loess hilly areas were significantly impacted by temperature. (*p* < 0.05, Figure 1). The germination rate and percentage of all species generally exhibited a tendency to rise, and then, fall with temperature (Figure 1). High or low temperatures inhibited the germination of different species to different degrees, and the germination percentage and rate of the studied seeds were higher at 15–30 °C than at 5–10 °C and 35 °C. For example, at 5 °C, only *Artemisia sacrorum* and *Periploca sepium* germinated, and the seeds of the other species did not germinate (Figure 1). Most species had low germination rates, except for some heat-tolerant plants such as *P. sepium* and *Linum usitatissmum* (Figure 1). The estimations of *T_b_*, *T_o_*, and *T_c_* were extrapolated using the linear relationship between germination rate and temperature. In addition, germination temperature thresholds were influenced by seed germination percentage (g) and varied with germination percentage between species (Figure 2, Table S1), making it difficult to determine a universal pattern. Linear increases and reductions in the germination rates below and above the *T_o_* were seen when the germination rates for various percentiles were plotted versus temperature. Specifically, *Lespedeza davurica* seeds could germinate in a variety of temperature ranges, whereas *Bothriochloa ischaemum*, *L. usitatissmum*, and *Sophora davidii* had a smaller range of germination temperatures and were more sensitive to temperature than the rest of the species. Figure 3 illustrates how the thermal time model may be used to estimate seed germination thresholds (Figure 3) more precisely. For most of the species under study, the goodness of fit between actual and anticipated germination rates was above 80% (Figure 3). The nine species were split into four distinct subgroups using heat maps or cluster analyses based on how the temperature affected the germination rate and germination percentage (Figure 4A). Specifically, one subgroup was *P. sepium*, which showed high tolerance for high temperatures, and another subgroup included *A. sacrorum*, which was more tolerant to cold than the rest of the species.

### 2.2. Germination Responses to the Water Potential

**Figure 5:**
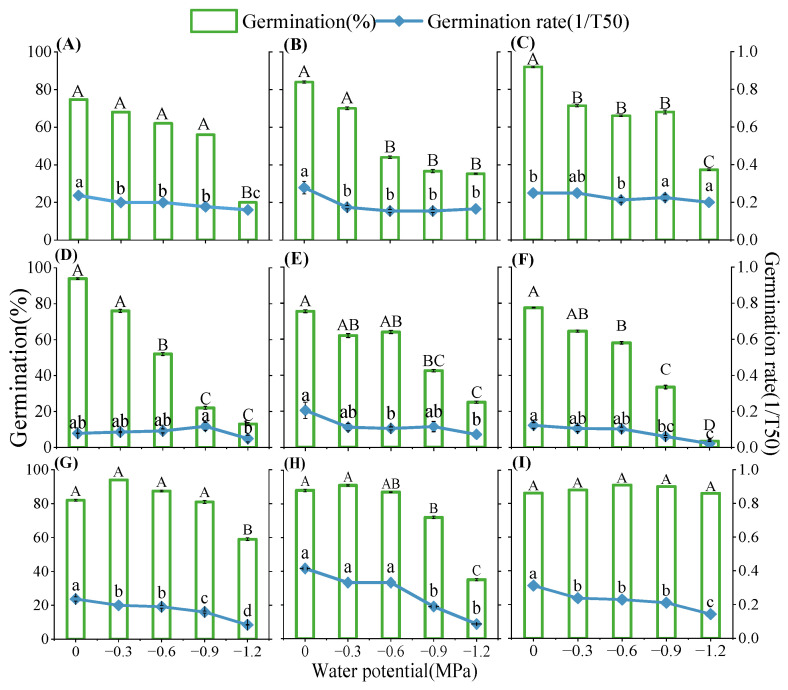
Percentage and rate (1/T_50_) of seed germination in nine species at five different water potential treatments; Different uppercase letters indicate that germination percentage differed significantly (*p* < 0.05) between water potential treatments; Different lowercase letters indicate that germination rate (1/T_50_) differed significantly (*p* < 0.05) between water potential treatments; (**A**) *Artemisia scoparia*, (**B**) *Artemisia giraldii*, (**C**) *Artemisia sacrorum*, (**D**) *Periploca sepium*, (**E**) *Bothriochloa ischaemum*, (**F**) *Patrinia scabiosifolia*, (**G**) *Linum usitatissimum*, (**H**) *Lespedeza davurica*, (**I**) *Sophora davidii*.

**Figure 6:**
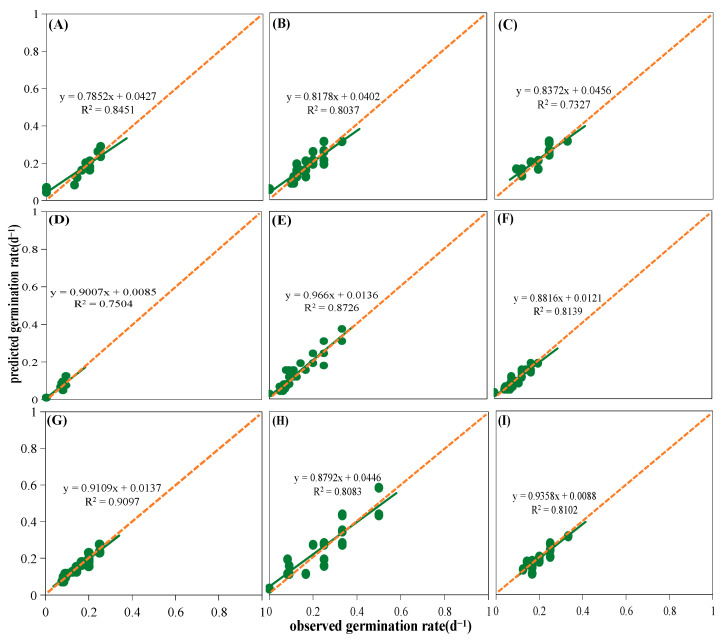
Linear fit of observed and predicted germination rates of nine species. The green line represents the fitted trend line of the observed germination rates to the predicted germination rates; the dots represent the predicted germination rates (predicted by the hydrotime model) for various observed germination rates. (**A**) *Artemisia scoparia*, (**B**) *Artemisia giraldii*, (**C**) *Artemisia sacrorum*, (**D**) *Periploca sepium*, (**E**) *Bothriochloa ischaemum*, (**F**) *Patrinia scabiosifolia*, (**G**) *Linum usitatissimum*, (**H**) *Lespedeza davurica*, (**I**) *Sophora davidii*.

Moisture had a significant impact on the germination percentages and rates of nine native plants of the loess hilly regions (*p* < 0.05, Figure 5). Germination percentages and rates decreased as water potential decreased; however, different plants responded to drought stress differently. For example, the germination percentage of *S. davidii* reached more than 80% under all five water potential treatments, while *Patrinia scabiosifolia*’s germination percentage and germination rate were close to 0 at −1.2 MPa (Figure 5). In addition, *S. davidii* and *L. usitatissmum* had a wide range of tolerance to drought stress, with their base water potentials lower than −2 MPa, while *P. sepium* had a very sensitive tolerance to the water potentials, with a base water potential of about −1.3 MPa (Table S2). Linear regression of the germination rate and water potential of the studied species revealed that the germination rate was significantly positively correlated with the water potential (Figure S1), and that the base water potential varied weakly with the germination rate (g) (Figure S1, Table S2). As shown in Figure 6, the hydrotime model was well fitted for all species, with a goodness of fit of more than 80% between observed and predicted germination rates for the vast majority of the studied species (Figure 6). Heat map or cluster analysis divided the nine species into three different subgroups according to the response of germination percentages and rates to water potential (Figure 4B). One subgroup was *P. scabiosifolia, B. ischaemum*, and *P. sepium*, which showed weak drought tolerance, and another subgroup was *A. sacrorum*, *Artemisia giraldii*, and *Artemisia scoparia*, which showed high drought tolerance (Figure 4B).

### 2.3. Germination Responses to the Interaction of Temperature and Water Potential

**Figure 7:**
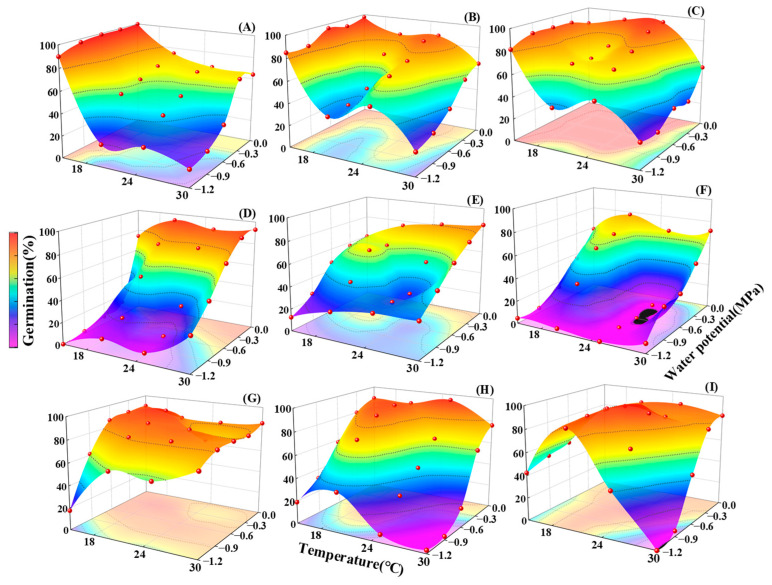
Interactions of temperature and water potential on the germination percentage of nine species; (**A**) *Artemisia scoparia*, (**B**) *Artemisia giraldii*, (**C**) *Artemisia sacrorum*, (**D**) *Periploca sepium*, (**E**) *Bothriochloa ischaemum*, (**F**) *Patrinia scabiosifolia*, (**G**) *Linum usitatissimum*, (**H**) *Lespedeza davurica*, (**I**) *Sophora davidii*.

Moisture and temperature are determinants of seed germination, and these two factors can individually or jointly affect germination percentage. The results of a two-way ANOVA on the studied species showed that the interaction of Species × *T*, Species × *Ψ*, *T* × *Ψ*, and Species × *T* × *Ψ* had a significant impact on the germination percentage (*p* < 0.001). At the same temperature, the germination percentage of most species decreased with decreasing water potential, but the response of different species to water potential varied with temperature. For example, the inhibitory effect of drought stress on germination in most species was more pronounced at 30 °C than at other temperatures (Figure 7). However, at 15 °C, drought stress appeared to more significantly inhibit the germination of the heat-tolerant *P. sepium*, *B. ischaemum*, and *L. usitatissmum* than that of the other species (Figure 7). In addition, temperature can modify the effect of water potential on the germination percentage; under the appropriate temperature, seeds can also achieve a high germination percentage under low water potential. For example, the germination percentage of *S. davidii* at −1.2 MPa also exceeded 80% at 20 °C (Figure 7). At all temperature levels, all species except *S. davidii* had the highest germination percentage at 0 MPa (Figure 7). Meanwhile, according to the analysis of the influences of temperature-and-water potential interactions on seed germination, the optimal temperature and moisture combinations were as follows: *A. scoparia*: 15 °C, 0 MPa; *A. giraldii*: 15 °C, 0 MPa; *A. sacrorum*: 25 °C, 0 MPa; *P. sepium*: 30 °C, 0 MPa; *B. ischaemum*: 30 °C, 0 MPa; *P. scabiosifolia*: 30 °C, 0 MPa; *L. usitatissmum*: 20 °C, −0.3 MPa; *L. davurica*: 25 °C, 0 MPa; *S. davidii*: 25 °C, −0.6 MPa.

### 2.4. Relationships between Germination Thresholds and Seed Traits

**Figure 8:**
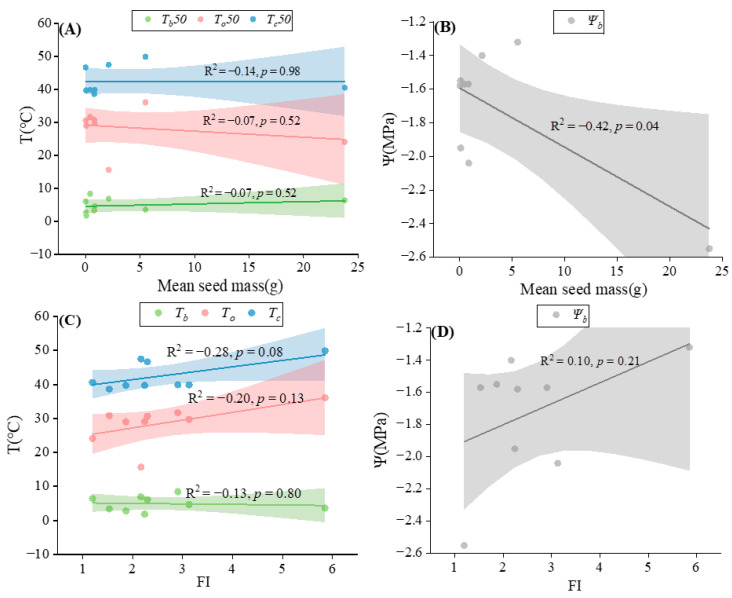
Linear fit of mean seed mass and flatness index (FI) of temperature thresholds, and water potential thresholds for nine species. (**A**) Mean seed mass and temperature thresholds; (**B**) mean seed mass and water potential thresholds; (**C**) FI and temperature thresholds; (**D**) FI and water potential thresholds.

The temperature thresholds for seed germination varied among the nine tested species (Table S1). Among them, the base temperature was the lowest in *P. scabiosifolia* and the highest in *B. ischaemum* (Table S1). A linear fit of mean seed mass to *T_b_* revealed a weak positive correlation (Figure 8A), whereas there was a weak negative correlation between FI and base temperature (Figure 8C). Among the tested species, the optimal germination temperature was the lowest in *L. davurica* and the highest in *P. sepium* (Table S1). Meanwhile, the optimal temperature was negatively correlated with mean seed mass and positively correlated with FI, but the correlation was not significant (*p* > 0.05, Figure 8A,C). The ceiling temperatures were lowest in *P. scabiosifolia* and highest in *L. davurica* (Table S1). Ceiling temperature was not correlated with mean seed mass, while there was a positive correlation with FI (Figure 8). The base water potential for germination also varied among species; it was highest in *P. sepium*, lowest in *S. davidii*, and ranged from −1.33 MPa to −2.07 MPa for the other species (Table S2). A significant negative relationship existed between base water potential and mean seed mass (*p* < 0.05, Figure 8B), and larger seeds had lower base water potentials than smaller seeds. In addition, the relationship between FI and base water potential was positive (Figure 8D), i.e., round seeds had lower base water potentials than elongated seeds.

## 3. Discussion

### 3.1. Effects of Temperature and Water Potential and Their Interaction in Seed Germination

Seed germination is an irreversible process, and once germination begins, the seedling is either self-perpetuating or dies [62]. The observation of seed radicle protrusion is a common method used to determine seed germination [63]. In many studies it is a widely accepted practice to use radicle protrusion greater than 2 mm as a criterion for determining seed germination and then calculating germination rates because this method offers a standardized and measurable indicator of seed viability and potential for successful growth. It is crucial to remember, nevertheless, that a radicle protrusion larger than 2 mm does not always imply the development of a seedling or fully grown plant [64]. For instance, poor circumstances like inadequate temperature, water, or nutrition availability may prevent a seed with a projecting radicle from developing into a viable seedling [65]. Temperature is an important factor affecting seed germination, and different plants germinate under different temperature ranges and optimal temperatures [55,66]. Temperature can speed up germination within a particular range, but extremes in temperature can hinder germination due to membrane permeability, membrane-binding activity, and the denaturation of enzymes [67,68]. The probable explanation is that suitable temperatures facilitate water uptake by the seed, enhance enzymatic processes and respiration, and store nutrients in a soluble state that is easy to use [69]. Understanding the response of plants to temperature not only helps us to understand their ecological adaptations, but also helps in developing effective vegetation restoration strategies [70]. This study’s findings on the species-specific responses of seed germination to temperature are in line with other research, as various species have different optimal temperatures and temperature ranges [71]. The different responses of plants to temperature reflect their degree of adaptation to the environment, and when plants are not well adapted to the local environment they will be eliminated by the environment [72]. In addition, the temperature threshold for seed germination in different species in the same habitat varies [15]. In this study, the thermal time model was used to derive the seed germination parameters of nine native species in the loess hilly region, and their base temperatures for germination ranged from −7.46 to 9.67 °C (Table S1). Among them, *P. scabiosifolia* exhibited seed germination at the lowest base temperature and could even germinate under a temperature below zero, and the cluster analysis also classified *P. scabiosifolia* as a subgroup (Figure 4A). Lower base temperature (*T_b_*) readings during germination indicate a plant’s ability to withstand cold stress [73]. In addition, compared to other species, *B. ischaemum*’s germination rate and germination percentage increased more quickly as the temperature rose, and a greater base temperature was required (Figure 1), which may be because *B. ischaemum* is a C4 plant and is light and warmth loving.

These findings demonstrated that the availability of water had an impact on the percentage and rate of seed germination. Water potential has been shown to have a significant negative influence on seed germination [26]. This germination inhibition may be thought of as a seed’s adaptive response to these circumstances; that is, the seeds will not germinate when exposed to lower *Ψ*, retaining their potential to germinate in order to reach the proper environmental conditions [29]. Distinct species have distinct hydrotime constants (*θ_H_*) and base water potentials (*Ψ_b_*), which affect how seeds germinate in response to water potential. The present study shows that seeds with low *Ψ_b_* germinated better than those with high *Ψ_b_* at a low water potential, which is consistent with Zhang et al. [34]. For example, *S. davidii* still had a germination percentage of more than 80% at −1.2 MPa, while *P. scabiosifolia* had a germination percentage close to 0 at −1.2 MPa. In addition, it is generally accepted that seeds that are suited to germinate in circumstances of water stress have an advantage when it comes to germination in arid or semiarid circumstances [74,75]. 

Numerous studies have found an interaction between *T* and *Ψ* regarding the percentage and rate of seed germination [76]. There was a positive correlation found between the temperature range and soil water availability of the plant in its natural environment and the capacity of seeds to germinate within that temperature range [77]. For example, tropical species often have greater *T_b_* requirements than temperate species, whereas mesic species are less resistant to water stress (i.e., can germinate at lower water potential) than tropical plants [78,79,80]. The species in this study, all of which are native to the loess hilly region, have evolved traits or strategies that enable them to resist local risks, such as the ability to germinate faster in hot environments to reduce the risk of drought. Our study shows that the germination time course of the examined seeds can be accurately described by the thermal time and hydrotime models, which is in line with other research conducted at different temperatures and water potentials, respectively [76,81]. Additionally, the two models and their standard deviation offer crucial details that are vital for both biology and ecology [82]. Recent years have seen a rise in the frequency of extreme temperature swings brought on by climate change, as well as erratic precipitation and protracted droughts [83,84]. In the context of climate change, evaluating the germination characteristics and resilience of native seeds in the loess hilly region and clarifying their suitable germination conditions are significant for assessing vegetation regeneration and ecological restoration in the region.

### 3.2. Ecological Correlates of Seed Performance

The germination requirements for temperature and moisture vary significantly across plant populations, and seed traits can drive plant community dynamics [85]. Studying the relationship between seed morphological characteristics, environmental factors, and seed germination helps us to predict ecological population dynamics [51,86]. This study showed that small seeds have a faster germination rate than large seeds under suitable temperature conditions (Figure 1). Since small seeds are less tolerant to stressful environmental conditions, they are more sensitive to temperature changes, allowing them to germinate rapidly and in large numbers under suitable conditions [87]. However, the present study showed that there was almost no correlation between temperature threshold and seed mass, which is inconsistent with previous studies [88]. The reason for this phenomenon may be that the temperature threshold is also influenced by other seed traits, such as seed coat thickness and permeability, which influence imbibition and germination [89,90]. Seed size showed a negative correlation with base water potential (Figure 8B), suggesting that larger seeds are more resistant to arid environments than small seeds. Seedlings raised from larger seeds are more resistant to drought, possibly because of their ability to explore larger amounts of soil to offset water scarcity, to build a more extensive root system, or to store more water [91,92]. Meanwhile, the high base water potential of small seeds is also a risk-reduction feature that results in seed germination in wetter conditions, which prevents seedlings from being exposed to dry conditions. The flatness index (FI) was positively correlated with optimal temperature, ceiling temperature, and base water potential, but none of these correlations were significant (Figure 8C,D). This may be because rounded seeds are more easily covered by soil, whereas flattened or elongated seeds are less likely to be covered by soil and are therefore more sensitive to the environment and subjected to greater stress [93].

### 3.3. Implications for Management and Conservation

Previous studies have demonstrated that vegetation restoration is the fundamental way to manage soil erosion in loess hilly regions [94]. However, the intra- and inter-annual temperature and precipitation variations in this region are drastic, which constrains the natural regeneration of seeds. Vegetation regeneration depends on seed germination, and there are interspecific differences in the response of germination to environmental variables [95]. Specifically, the cluster analysis showed that *P. sepium* was more tolerant to high temperatures than the other species. This may be because *P. sepium* is the species at the highest stage in the succession and can adapt to high-light habitats, so it could be considered as a species for vegetation restoration on sunny slopes in the loess hilly region. In general, large seeds are more tolerant to arid environments, so resowing large seeds rather than small seeds can be considered as a method of vegetation restoration under poor moisture conditions. Furthermore, as vegetation succession proceeds, habitat differentiation in vegetation types occurs, mainly in the upward slope direction; for example, *A. giraldii* is mainly distributed on sunny slopes, while *A. sacrorum* is mainly distributed on shady slopes [96]. Depending on the impacts of temperature and water potential on native species, it is feasible to select seasons and microhabitats (e.g., slope orientation, slope position) that are suitable for their emergence; regarding environments with harsher thermal and hydrological conditions, it is advisable to consider selecting more tolerant species for sowing to promote vegetation regeneration. Land managers should understand the seed germination needs of different populations to assure that seeds are planted at the most conducive times and under the most favorable conditions to accomplish vegetation restoration [18].

## 4. Materials and Methods

### 4.1. Study Site

**Figure 9:**
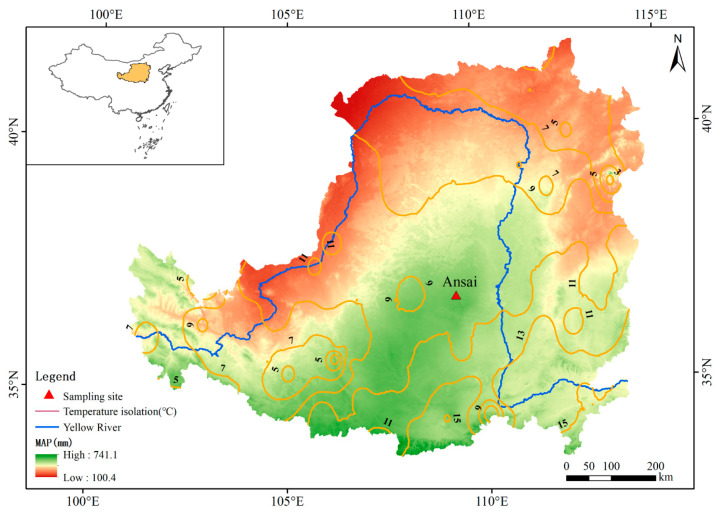
Map showing the location of the study site.

The study area, Ansai, is situated in the northwest of Yan’an City, Shaanxi Province, China (109°16.7654′ E–109°17.3011′ E, 36°44.1054′ N–36°48.0715′ N), with an average altitude of 1371.9 m a.s.l. (Figure 9), and it is a typically hilly and gullied region of the Loess Plateau. The annual average temperature is about 9.8 °C, the frost-free period is about 180 days, the annual precipitation is about 660 mm, and the annual average land evaporation is about 1460 mm. The soil is mainly loess soil, with 33.2% sand, 63.2% silt, and 3.6% clay [97]. The type of vegetation belongs to the forest-steppe zone, and the dominant species are *Bothriochloa ischaemum*, *Stipa bungeana*, *Periploca sepium*, etc. The area has 58% vegetation coverage [98].

### 4.2. Seed Collection and Preparation

This study used nine native plant species with high cover from the hilly–gullied Loess Plateau region, representing the most common species used for vegetation restoration succession in the region and belonging to different families with different seed traits (size, shapes, etc.). Seeds were collected in 2019 in the small watershed of Zhifanggou and Fangta, Ansai District, Yan’an City, and stored under dry, ventilated chamber conditions for one year. The germination experiments were started in September 2020 and the sampled seeds were sterilized in 10% sodium hypochlorite solution (Shanghai, China) for 10 min and rinsed with deionized water for 5 min before the experiment. The seeds were then placed on germination medium and a viability test was carried out prior to the germination test. The germination percentage of the seeds in distilled water at room temperature was more than 80%, indicating that the seeds tested in this experiment were non-dormant [99]. We mechanically cut *S. davidii* and *L. davurica* seeds with a scalpel, stripping the seed coat to make them permeable to water. The seed mass was measured using a sensitive balance (over 1/10,000 level) (Shanghai, China) (Table 1) [100]. Meanwhile, the length (L), width (W), and height (H) of the seeds were measured using a slide caliper with four replicates, and the FI (FI = (L+ W)/2 H) was used to characterize the seed shape [101]. The FI ranged from a value of 1 for spherical seeds to greater values for seeds with flat or spindle shapes [93,102].

### 4.3. Experimental Design

#### 4.3.1. Temperature Effects

In this experiment, seven fixed temperatures—5, 10, 15, 20, 25, 30, and 35 °C—were utilized. A germination test was conducted with four replications by placing the seeds in Petri (Yangzhou, China) dishes measuring 90 mm in diameter on two sheets of filter paper (Hangzhou, China) saturated with 10 mL distilled water. Then, the Petri dishes were placed in a thermostatic incubator (Shanghai, China) for germination, which was programmed for a 12 h photoperiod under 0 MPa. To avoid seed germination competition due to higher density or number, we set the quantity of *P. sepium* and *L. davurica* seeds to 25 seeds; the larger seeds of *S. davidii* to 20; and the rest of the seeds to 50, according to their length, width, and height. The quantity of seeds that germinated was tallied every day, and germination was defined as when the radicle broke through the seed coat and the length of the radicle was ≥2 mm. A germination period of 30 days was used, and when there was no more germination after three consecutive days, the germination test was terminated.

#### 4.3.2. Water Potential Effects

After the end of the temperature experiment, we found that most of the seeds exhibited better germination performance at 20 °C. Therefore, we chose to evaluate the influence of water potential on germination under the temperature condition of 20 °C. In Petri dishes measuring 90mm in diameter, four seed duplicates were arranged on two filter paper sheets wetted with either a separate 10 mL PEG solution or distilled water (control). Solutions of polyethylene glycol 6000 (PEG 6000) (Hangzhou, China) were made in accordance with Michel and Kaufmann’s (1973) protocols [103]. Every other day, the seeds were moved to new filter paper that contained either distilled water or a fresh solution in order to maintain relatively steady water potential during germination. The seed number settings for the species were consistent with those of the temperature-effect germination experiments. A light (12 h/12 h, daily photoperiod) was used to incubate the seeds at 20 °C with varying water potentials of −0.3, −0.6, −0.9, and −1.2 MPa. The number of germinated seeds was counted every day, and the germination period was 30 days.

#### 4.3.3. Interactions of Temperature and Water Potential

By analyzing the temperature experiments, we found that the studied seeds had a higher germination rate between 15 and 30 °C. Therefore, this experiment set four constant temperatures (15, 20, 25, and 30 °C) and five levels of water potential (0, −0.3, −0.6, −0.9, and −1.2 MPa), resulting in twenty different treatments, to investigate the combined influence of temperature and water potential on seed germination. The number of seeds and the incubator condition settings were consistent with the above two germination experiments. The number of germinated seeds was counted every day and the germination period was 30 days.

### 4.4. Mathematical Models

To calculate the time process of germination changing with temperature, a thermal time model was used [42]. This model can be written as

*θ_T(g)_* = (*T* − *T_b_*)*t_g_* (1)

or

GR_g_ = 1/*t_g_* = (*T* − *T_b_*)/*θ_T(g)_* (2)

*θ_2_* = (*T_c(g)_* − *T*)*t_g_* (3)

or

GR_g_ = 1/*t_g_* = (*T_c(g)_* − *T*)*t_g_*/*θ*_2_ (4)

The temperature (°C), base temperature (°C), time to achieve a specific germination percentage g (d), thermal time constant for a specific germination rate (g) in the population at a sub-optimal temperature (°C·d), thermal time constant at supra-optimal temperatures (°C·d), and ceiling temperature (°C) are represented by *T*, *T_b_*, *t_g_*, *θ_T(g)_*, *θ*_2_, and *T_c(g)_*, respectively [29,42].

A hydrotime model (HT; Equations (5) and (6)) proposed by Gummerson (1986) was used to quantify the response of germination rate to *Ψ* [104]:

*θ_H_* = (*Ψ* − *Ψ_b(g)_*)*t_g_* (5)

or

GR_g_ = 1/*t_g_ =* (*Ψ* − *Ψ_b(g)_*)/*θ_H_* (6)

The hydrotime constant (MPa·h), real water potential (MPa), and base water potential (MPa) or drought tolerance threshold values are represented by the variables *θ_H_*, *Ψ*, and *Ψ_b(g)_* [10,104].

The degree of fit between the predicted and observed data was evaluated using the coefficient of determination (*R^2^*). The fraction variance accounted for by the simulation model *R^2^* was calculated using Equation (7):

(7) $$R^{2}=1-\sum {\left(y_{\mathrm{obs}}-y_{\mathrm{pre}}\right)}^{2}/\sum {\left(y_{\mathrm{obs}}-{\overset{-}{y}}_{\mathrm{obs}}\right)}^{2}$$

where y_obs_ represents the observed values, y_pre_ represents the predicted values, and ${\overset{-}{y}}_{\mathrm{obs}}$ represents the mean of the observed values. An *R*^2^ value of 1 indicates that the model is in perfect accordance with the observations.

### 4.5. Statistical Analysis

All statistical analyses were conducted using SPSS 22.0, Excel 2021, and Origin 9.1 software. In both experiments, we employed one-way ANOVA to investigate the effects of temperature and water potential on the germination percentage, or rate (1/T50), for each species. The effects of species, temperature, and water potential on germination characterizations were tested using two-way ANOVA. The Duncan test was used to screen for significant differences between treatments at *p* < 0.05. The K-means clustering approach was utilized to determine clusters of distinct subclasses. We used a thermal time model to determine the temperature threshold for species germination and a hydrotime model to determine the base water potential for the germination of different species. Linear fitting between the observed and predicted germination rates was performed, and the coefficient of determination (*R*^2^) was used to evaluate the goodness of fit of the model. Meanwhile, the association between seed morphological characteristics and plant responses to temperature and water potential was evaluated through the linear fitting of seed size and shape to base temperature and base water potential, respectively.

## 5. Conclusions

Our findings show that in most species, the percentage and pace of germination increased initially, declined with rising temperature, and subsequently dropped with falling water potential. The species had different temperature thresholds and water potential thresholds, and the thresholds changed with increasing germination percentage. In general, the base water potential for germination was negatively correlated with mean seed mass and was lower for more rounded seeds than for elongated seeds. However, a general mechanism cannot yet be generalized between temperature thresholds and either mean seed mass or FI. Each species differed in their resistance to external environmental stresses; among them, *A. sacrorum* was more resistant to low temperatures; *P. sepium* to high temperatures; and *L. davurica*, *S. davidii*, *L. usitatissmum*, *A. sacrorum*, *A. giraldii*, and *A. scoparia* to drought stresses. Plants with strong resistance to high temperatures and drought can be prioritized as species for vegetation restoration in loess hilly regions. The seed germination of the nine native plant species can be accurately predicted using both the thermal and hydrotime models, which can be used to forecast how the region’s flora will spread in the event of climate change. However, the limitations of this study are the relatively small number of species selected and the fact that only the effects of seed size and shape on germination thresholds were considered. Future studies should increase the number of species and consider the relationship between other seed traits (e.g., appendages and seed coat) and germination thresholds to achieve a comprehensive understanding of the limiting factors of vegetation regeneration in the region under climate change, and also to provide a theoretical basis for ecological restoration construction.

## Figures and Tables

**Table 1:** Seed morphology of the experimental species.

Species	Family	Length (mm)	Width (mm)	Height (mm)	Individual Mass (g)	Shape	Appendages	FI	Seed Storage Time (Month)
*Artemisia scoparia*	Asteraceae	0.662 ± 0.035	0.325 ± 0.019	0.215 ± 0.013	0.020	Oval-circular	None	2.295	12
*Artemisia giraldii*	Asteraceae	0.923 ± 0.024	0.420 ± 0.018	0.360 ± 0.020	0.061	Oval	None	1.865	12
*Artemisia sacrorum*	Asteraceae	1.091 ± 0.048	0.477 ± 0.014	0.350 ± 0.030	0.085	Oval	None	2.240	12
*Periploca sepium*	Apocynaceae	8.152 ± 0.068	1.820 ± 0.050	0.852 ± 0.009	5.506	Long-circular	Hair	5.852	12
*Bothriochloa ischaemum*	Poaceae	1.986 ± 0.085	0.744 ± 0.030	0.470 ± 0.036	0.432	Long-spindle	Awn	2.904	12
*Patrinia scabiosifolia*	Caprifoliaceae	2.244 ± 0.029	1.156 ± 0.024	1.110 ± 0.037	0.810	Ellipsoid	Wing	1.532	12
*Linum usitatissimum*	Linaceae	2.722 ± 0.106	1.446 ± 0.083	0.666 ± 0.064	0.849	Long-oval	None	3.129	12
*Lespedeza davurica*	Fabaceae	3.238 ± 0.185	1.770 ± 0.053	1.156 ± 0.038	2.129	Obovate	None	2.166	12
*Sophora davidii*	Fabaceae	3.064 ± 0.038	3.992 ± 0.087	2.952 ± 0.047	23.769	Oval	None	1.195	12

## Data Availability

Data are contained within the article and Supplementary Materials.

## Supplementary Materials

The following supporting information can be downloaded at: https://www.mdpi.com/article/10.3390/plants13050693/s1, Figure S1: Linear regression of germination rate (1/tg) of nine species (different percentiles) and water potentials; Table S1: Estimation of cardinal temperatures for seed germination of nine species at 0 MPa using linear regression analysis at different percentiles (20–80%); Table S2: Estimation of cardinal water potential for seed germination of nine species at 20 °C using linear regression analysis at different percentiles (20–80%).
