# Supplementary material for: The Impact of Water Potential and Temperature on Native Species’ Capability for Seed Germination in the Loess Plateau Region, China
Source: Plants (Basel). 2024 Feb 29;13(5):693. doi: 10.3390/plants13050693 (PMC10934695; doi:10.3390/plants13050693)
Supplement: Supplementary file 1 [file plants-13-00693-s001.zip › plants-2870061-supplementary.pdf]

1 Supplementary Figures and Tables

1.1 Supplementary Figures

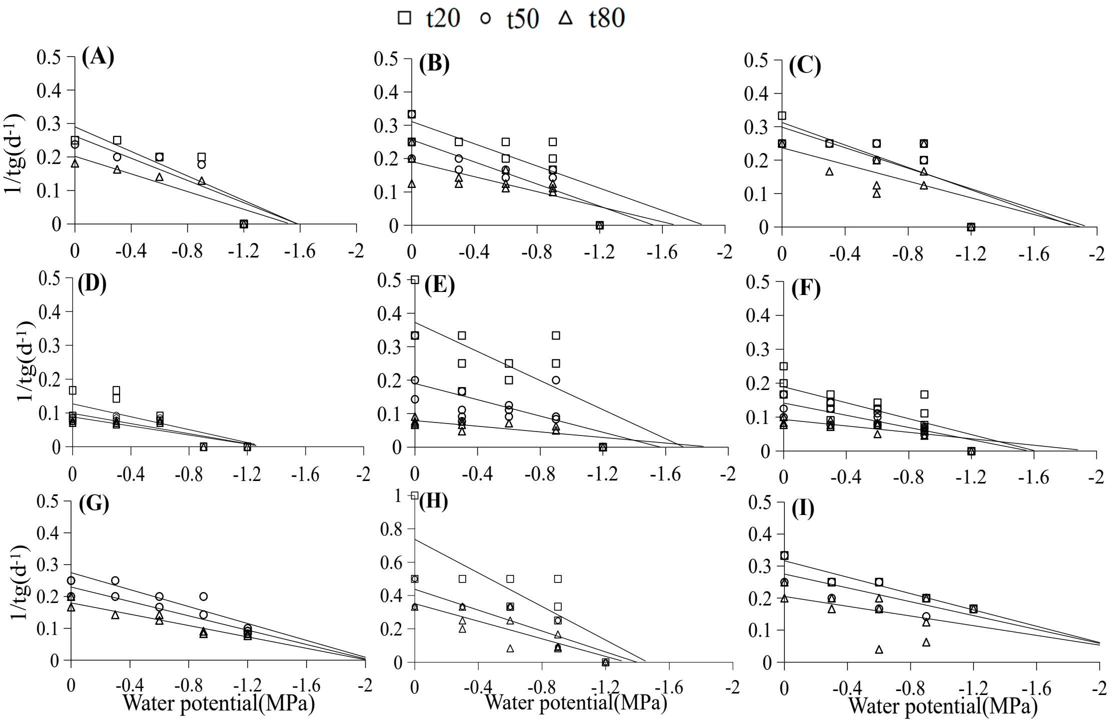

Supplementary Figure S1. Linear regression of germination rate (1/tg) of nine species (different percentiles) and water potentials; (A) *Artemisia scoparia*, (B) *Artemisia giraldii*，(C) *Artemisia sacrorum*, (D) *Periploca sepium*, (E) *Bothriochloa ischaemum*, (F) *Patrinia scabiosifolia* (G) *Linum usitatissimum*, (H) *Lespedeza davurica*, (I) *Sophora davidii*.

1.2 Supplementary Tables

Supplementary Table S1 Estimation of cardinal temperatures for seed germination of nine species at 0MPa, using a linear regression analysis at different percentiles (20-80%).

Species	percentiles	T_b(°C)	T_o(°C)	T_c(°C)	Species	percentiles	T_b(°C)	T_o(°C)	T_c(°C)	Species	percentiles	T_b(°C)	T_o(°C)	T_c(°C)
Artemisia scoparia	20	6.23	30.76	45.05	*Artemisia giraldii*	20	3.35	29.58	39.52	*Artemisia sacrorum*	20	3.40	29.81	39.52
	30	5.86	31.45	45.05		30	3.90	29.38	40.15		30	2.35	29.15	40.15
	40	5.43	30.52	46.67		40	4.08	29.71	40.15		40	2.43	29.35	40.15

	50	6.14	30.61	46.67		50	2.80	29.01	39.76		50	1.85	29.12	39.76
	60	6.36	31.30	46.67		60	2.26	29.42	43.05		60	1.57	29.25	38.07
	70	4.70	31.66	49.90		70	2.98	29.27	38.24		70	1.31	28.98	38.24
	80	4.54	31.16	47.14		80	2.52	27.32	40.85		80	1.09	29.96	40.30
Periploca sepium	20	6.65	32.86	42.54	*Bothriochloa ischaemum*	20	6.67	30.27	40.45	*Patrinia scabiosaefolia*	20	3.53	29.20	38.64
	30	4.51	34.00	49.90		30	7.46	31.85	40.00		30	3.94	29.92	38.64
	40	4.64	34.51	49.90		40	7.17	32.51	38.48		40	2.89	31.27	38.64
	50	3.63	36.10	49.90		50	8.44	31.68	39.89		50	3.39	30.79	38.63
	60	3.39	36.73	49.90		60	8.18	31.59	39.08		60	4.05	31.20	38.63
	70	3.83	34.82	46.00		70	8.93	33.87	41.60		70	1.37	30.44	39.75
	80	1.73	34.56	49.15		80	9.67	32.52	41.73		80	-7.46	30.46	41.41
Linum usitatissmum	20	4.34	29.98	40.29	*Lespedeza davurica*	20	6.44	19.26	41.85	*Sophora davidii*	20	6.35	24.05	42.11
	30	4.62	29.82	40.04		30	6.93	16.75	48.03		30	6.66	24.05	40.62
	40	4.88	29.72	39.86		40	6.94	15.65	47.68		40	6.64	24.18	40.55
	50	4.67	29.71	39.86		50	6.96	15.67	47.48		50	6.46	24.09	40.55
	60	4.71	30.11	39.73		60	7.37	15.28	46.43		60	6.47	24.28	40.37
	70	5.28	30.20	39.55		70	7.24	15.17	52.40		70	6.25	24.33	40.37
	80	3.86	29.12	40.22		80	6.50	15.81	61.96		80	6.11	24.26	40.68

Supplementary Table S2 Estimation of cardinal water potential for seed germination of nine species at 20°C, using a linear regression analysis at different percentiles (20-80%).

Species	percentiles	Ψ_b (MPa)	Species	percentiles	Ψ_b (MPa)	Species	percentiles	Ψ_b (MPa)
Artemisia scoparia	20	-1.58	*Artemisia giraldii*	20	-1.87	*Artemisia sacrorum*	20	-1.86
	50	-1.58		50	-1.55		50	-1.95
	80	-1.53		80	-1.69		80	-1.90
Periploca sepium	20	-1.33	*Bothriochloa ischaemum*	20	-1.72	*Patrinia scabiosaefolia*	20	-1.61
	50	-1.32		50	-1.57		50	-1.57
	80	-1.32		80	-1.89		80	-1.96
Linum usitatissmum	20	-2.07	*Lespedeza davurica*	20	-1.46	*Sophora davidii*	20	-2.48
	50	-2.04		50	-1.40		50	-2.55
	80	-2.02		80	-1.33		80	-2.71
